# The significance of partial volume effect on the estimation of hypoxic tumour volume with [^18^F]FMISO PET/CT

**DOI:** 10.1186/s40658-024-00643-1

**Published:** 2024-05-09

**Authors:** Athanasios Kafkaletos, Michael Mix, Ilias Sachpazidis, Montserrat Carles, Alexander Rühle, Juri Ruf, Anca L. Grosu, Nils H. Nicolay, Dimos Baltas

**Affiliations:** 1 Division of Medical Physics, Department of Radiation Oncology, Medical Center – University of Freiburg, Faculty of Medicine, German Cancer Consortium (DKTK), partner site DKTK-Freiburg, University of Freiburg, Freiburg, Germany; 2 Department of Radiation Oncology, Medical Center – University of Freiburg, Faculty of Medicine, German Cancer Consortium (DKTK), partner site DKTK-Freiburg, University of Freiburg, Freiburg, Germany; 3 Department of Nuclear Medicine, Medical Centre - University of Freiburg, Faculty of Medicine, German Cancer Consortium (DKTK), partner site DKTK-Freiburg, University of Freiburg, Freiburg, Germany; 4 German Cancer Consortium (DKTK), Partner Site Freiburg, a partnership between DKFZ and Medical Center – University of Freiburg, Freiburg, Germany; 5 Biomedical Imaging Research Group (GIBI230-PREBI) and Imaging La Fe Node at Distributed Network for Biomedical Imaging (ReDIB) Unique Scientific and Technical Infrastructures (ICTS), La Fe Health Research Institute, Valencia, Spain; 6 Department of Radiation Oncology, University of Leipzig Medical Centre, Leipzig, Germany

**Keywords:** Hypoxia, Partial volume effects, PET, FMISO, HNSCC

## Abstract

**Background:**

The purpose of this study was to evaluate how a retrospective correction of the partial volume effect (PVE) in [^18^F]fluoromisonidazole (FMISO) PET imaging, affects the hypoxia discoverability within a gross tumour volume (GTV). This method is based on recovery coefficients (RC) and is tailored for low-contrast tracers such as FMISO. The first stage was the generation of the scanner’s RC curves, using spheres with diameters from 10 to 37 mm, and the same homogeneous activity concentration, positioned in lower activity concentration background. Six sphere-to-background contrast ratios were used, from 10.0:1, down to 2.0:1, in order to investigate the dependence of RC on both the volume and the contrast ratio. The second stage was to validate the recovery-coefficient correction method in a more complex environment of non-spherical lesions of different volumes and inhomogeneous activity concentration. Finally, we applied the correction method to a clinical dataset derived from a prospective imaging trial (DRKS00003830): forty nine head and neck squamous cell carcinoma (HNSCC) cases who had undergone FMISO PET/CT scanning for the quantification of tumour hypoxia before (W0), 2 weeks (W2) and 5 weeks (W5) after the beginning of radiotherapy. Here, PVE was found to cause an underestimation of the activity in small volumes with high FMISO signal.

**Results:**

The application of the proposed correction method resulted in a statistically significant increase of both the hypoxic subvolume (171% at W0, 691% at W2 and 4.60 × 10^3^% at W5 with *p* < 0.001) and the FMISO standardised uptake value (SUV) (27% at W0, 21% at W2 and by 25% at W5 with *p* < 0.001) within the primary GTV.

**Conclusions:**

The proposed PVE-correction method resulted in a statistically significant increase of the hypoxic fraction (HF) with *p* < 0.001 and demonstrated results in better agreement with published HF data for HNSCC. To summarise, the proposed RC-based correction method can be a useful tool for a retrospective compensation against PVE.

## Introduction

The motivation for this study was the need for an accurate and robust method to locate and quantify tumour hypoxia in head and neck squamous cell carcinoma (HNSCC). The main treatment modalities for locoregionally advanced HNSCCs are either surgical resection with risk-adapted postoperative (chemo)radiation or definitive radiotherapy in combination with concomitant systemic treatment[1–5]. The anti-tumour efficacy of radiotherapy is inversely correlated with the presence of hypoxia in HNSCCs [6–11]. The tumour-associated hypoxia can be non-invasively monitored by positron emission tomography (PET) imaging, using hypoxia-PET-tracers, the most widespread being [^18^F]fluoromisonidazole (FMISO) [12].

The partial volume effect (PVE) in positron emission tomography (PET), a consequence of the limited spatial resolution of PET-imaging, is a well-known obstacle in quantitative analysis of PET-imaging, leading to underestimation of activity concentration in tissues of small volume and higher activity concentration than surrounding tissues (background, BG). PVE has been extensively analysed and there have been many attempts to establish adequate correction methods [13–18]. The majority of those methods require post-processing of raw data and/or complementary imaging data and generate either regional or global image corrections.

PVE correction is not generally available for PET/CT scanners currently in clinical use. The PET/CT scanner, which all our clinical data were obtained from, did not offer PVE correction. So, the aim of this study was to establish a method to retrospectively compensate for the PVE in FMISO PET/CT, and thus to enable an improved quantitative analysis of the available clinical PET data regarding tumour hypoxia.

The FMISO tracer was used to locate hypoxic regions inside the gross tumour volume (GTV) and the quantification of hypoxia was addressed only within the GTV. A commonly used threshold of the standardised uptake value (SUV) was implemented in this study to define the hypoxic tumour volume (HTV) within GTV [19–26].

In order to address the challenges posed by PVE, we explored published image-based correction techniques. Given the study's focus on hypoxia within a specific region of interest, namely the GTV, we selected an established, region-based method and adapted it to align with the unique requirements and characteristics of our investigation, especially with the low contrast of FMISO. The recovery coefficient (RC)-based correction method [27, 28] is region-based and can be applied retrospectively to reconstructed PET images without the requirement of raw data or supplementary images from different modalities. So, based on the aforementioned facts and since raw data from the PET-CT acquisitions were not available for our investigation, the RC-based method was considered as most suitable for the PVE correction.

PVE is scanner-specific and thus a dedicated RC-model had to be developed based on experimental measurements at the PET scanner used for the underlying clinical trial. First, the RCs were calculated based on experimental measurements using a phantom with homogeneous, high activity (hot) spheres of different volumes, in lower activity (warm) background. RCs in dependence on volume and contrast were extracted and an analytic model was fitted to these results. Specifically for this project, RC was analytically modelled to describe both contrast and volume dependence and to include low contrast levels, as low as 2:1, typical for FMISO imaging. In a next step, the developed RC-model for PVE correction was validated using PET acquisitions of a dedicated phantom set-up containing several cylindrical elements of inhomogeneous activity distribution. Finally, the validated PVE correction model was applied retrospectively to patient FMISO PET data.

## Materials and methods

### FMISO PET/CT measurements design for Partial Volume Effects correction

#### Description of the PET/CT device

The measurements for the RC calculation and validation, as well as all of the clinical PET/CT image acquisitions were done on a Philips Gemini TF BigBore 16 PET/CT (Eindhoven, The Netherlands) at the Nuclear Medicine department at Medical Centre University Freiburg. It is a full three-dimensional, time-of-flight capable whole-body PET scanner, combined with a 16-slice Brilliance CT scanner unit. The PET scanner is designed with a 90 cm scanner diameter and an 18 cm axial field of view. It uses flat modules of a 23 × 44 array of 4 × 4 × 22 mm^3^ discrete lutetium-yttrium oxyorthosilicate (LYSO) crystals in a pixelated Anger-logic detector design arrangement. The spatial resolution in both the transverse and the axial directions is 4.8 mm near the central axis [29]. The scanner uses an iterative reconstruction algorithm with spherical coordinates (BLOB-OS-TF), 3 iterations, 33 subsets and a relaxation parameter for smoothing of 0.35. The voxel size of the reconstruction is isotropic at 2 mm. Finally, correction for random- and scatter-coincidences and photon attenuation is provided, based on the combined CT scan [30].

#### Description of the phantoms

##### Phantom-1

**Fig. 1:**
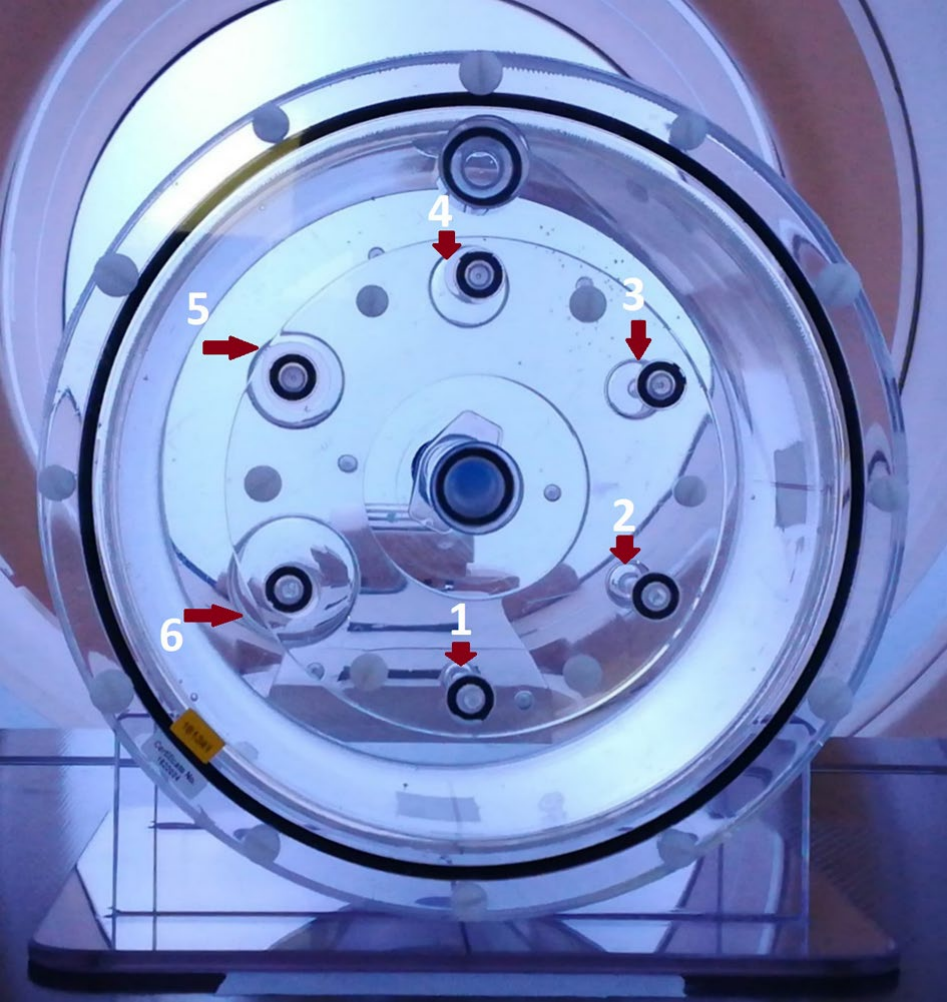
The cylindrical phantom used for the first stage of the development of the correction model from spherical lesions with homogeneous activity concentration. The six spheres are numbered from 1 to 6 with increasing volume

The phantom set-up used for the calculations of the RC curves (Fig. 1) was the PTW PET/SPECT-phantom, set T43004.1.008-0106 (PTW, Freiburg, Germany). The phantom body is cylindrical with an inner diameter of 200 mm and an outer diameter of 236 mm made of polymethylmethacrylate (PMMA) [31]. The phantom includes an insert, which has six hollow glass spheres with inner, active diameters of 10, 13, 17, 22, 28, and 37 mm.

The aim was to have a set-up that resembles the conditions of the head and neck region of the human body during FMISO PET/CT acquisition in terms of activity and contrast of the HTV against its surrounding tissues (BG). We repeated the measurements for six contrasts (10.0:1, 7.6:1, 5.8:1, 4.0:1, 2.5:1, 2.0:1) of hot (high activity) spheres against warm (lower activity) BG and used water for the representation of the BG and the HTV because it resembles soft tissue density. For all image acquisitions, we used the same imaging protocol that was used in the clinical study.

##### Phantom-2

**Fig. 2:**
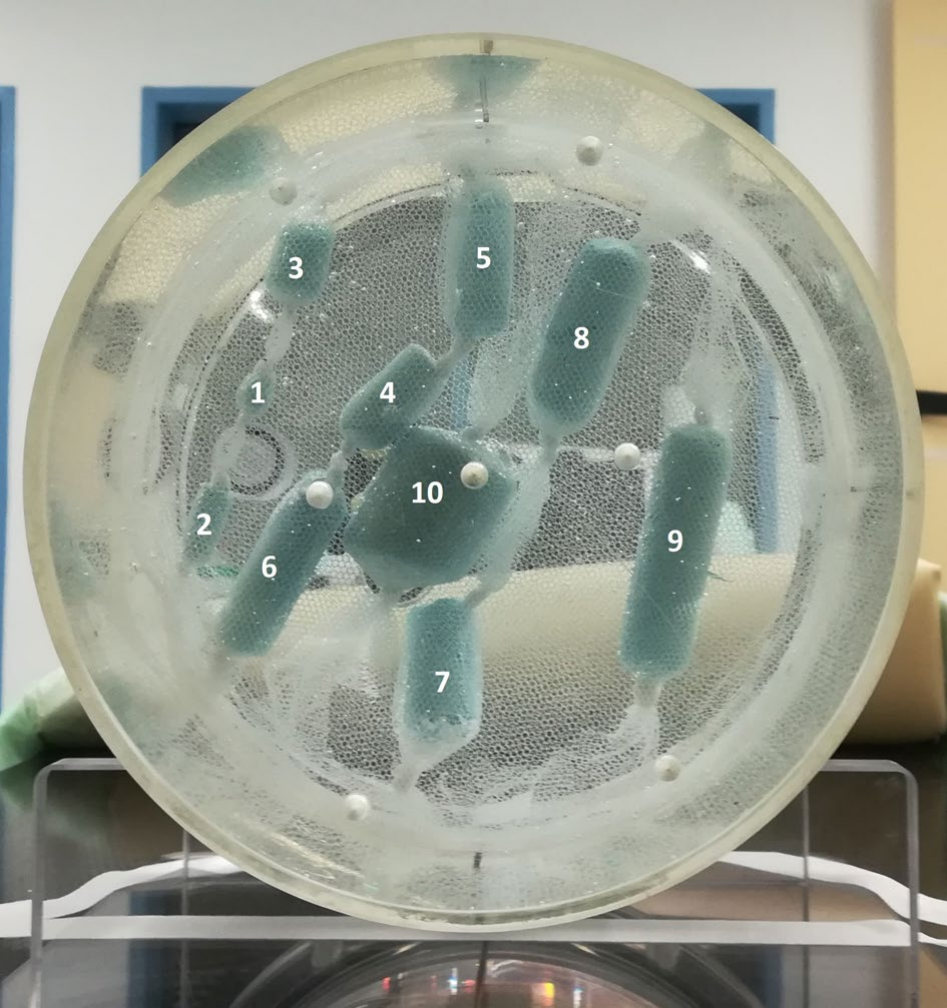
The cylindrical phantom used for the validation of the PVE correction model on non-spherical lesions with inhomogeneous activity distributions. There are ten blue cylinders, suspended in water with a net that is stabilised on the phantom’s walls with Velcro straps. The cylinders are numbered with increasing size and the seven largest (4–10) are used for the analysis

The phantom set-up that was used for the validation of the RC correction method (Fig. 2) has the same body as phantom-1 but instead of the glass spheres, we inserted custom-made cylinders of different volumes. For the construction of the cylinders, we used alginate, mixed with a solution of [^18^F]fluordesoxyglucose (FDG). Each cylinder contained alginate of two different activity concentrations with a contrast of 1.3:1, resembling the contrast SUV_mean_:SUV_max_ in the HTV of the FMISO patients of the study, and they were all submerged in water of lower activity [32]. The set up was scanned at three lesion-to-background contrasts, starting with the highest (7:1) and proceeding with introduction of extra activity to the background, to decrease the contrast twice (4:1 and 2:1).

#### Calculation of recovery coefficient

In this study, the RC was used for the PVE correction. The PET scanner measures only a portion of the actual activity in small volumes, when they are located in a background of lower activity. This portion depends both on the size of the lesion and on the local contrast.

Considering the geometry in phantom-1 (Fig. 1), the contours of the spheres were delineated on the CT image from the phantom's PET/CT scan and then, they were transferred to the PET image. Due to the lower spatial resolution of the PET, in some cases, the contours of the smallest spheres were not accurately replicated on the PET image. Consequently, manual adjustments were performed to correct these inaccuracies in the contours on the PET image.

The RC for a specific sphere, filled with a tracer solution of known activity concentration, is defined by the following equation [17, 18]:

1 $$RC = \frac{measured \;sphere \;activity\; concentration}{{actual \;sphere \;activity \;concentration}}$$

The dependence of RC on volume was measured using phantom-1 for the embedded six spheres of different sizes for a specific contrast level.

Although, PVE depends on the dimensions and not directly on the volume of the region of interest (ROI) [13, 14], in our experimental set-up with spherical ROIs both volume and radius/diameter dependence of RC were equivalent. Using the semi-logarithmic representation of the RC values in relation to volume, data could be modeled for each contrast level [27]:

2 $$RC = a \times ln\left( V \right) + b$$

The linear regression fit was done using MATLAB (R2020b, Update 2, Natick, Massachusetts: The MathWorks Inc.).

The above model demonstrated equivalent performance in fitting our experimental data at different contrast levels with the also investigated two-parameter logistic function, as presented by Gear et al. [18], when focusing in the range of volume and contrast that were used in our investigation. The semi-logarithmic model was finally preferred since it could be easily expanded to describe the contrast dependence of RC.

#### Contrast dependence of RC

In published investigations, the dependence of RC on contrast for a specific volume was addressed by, either a nearest neighbour [27] or a look-up-table-based [28] correction approach. In the present work and based on our measurements using the phantom-1 at six different contrast levels, we considered explicitly both volume and contrast dependence of RC. For that purpose and based on Eq. 2, RC can be written as a function of both volume (*V*) and contrast (*C*):

3 $$RC\left( {V,C} \right) = a\left( C \right) \times ln\left( V \right) + b\left( C \right)$$

The dependence of both *a*(C) and *b*(C) coefficients on contrast was determined by fitting Eq. 2 to the experimental RC data for the six different contrast levels used in our experiments.

#### Validation with inhomogeneous activity volumes

The introduced correction method is validated in a more complex and realistic set-up using the phantom-2 (Fig. 2) with inhomogeneous activity concentrations. The generalised RC model (Eq. 3) was applied on the measured activity values of the seven cylinders in phantom-2 by multiplying the measured activity with the inverse of RC [16].

Two different methods were considered to define the appropriate RC value for each of the seven cylinders: (a) The volume of each cylinder is directly used in Eq. 3 (volume-method) and (b) the diameter of the cylinder, that is the smallest dimension of the ROI, is used to calculate the volume of a corresponding sphere to be used in Eq. 3 (diameter-method).

### Application of PVE correction to patient data

#### Patient cohort

Our cohort consisted of forty-nine HNSCC patients treated with definitive chemoradiation treatment (CRT) at the University Medical Centre Freiburg within a prospective imaging trial. The trial received approval by the Independent Ethics Committee of the University of Freiburg (reference no. 479/12) and was performed according to the Declaration of Helsinki (revised version of 2008). It is registered at the German Clinical trials Register (DRKS00003830). CRT was administered for 7 weeks in daily fractions of 2 Gy to a total dose of 70 Gy to the primary tumour and macroscopic lymph node metastases and 50 Gy to the elective lymphatic drainage [33]. The Sequential Boost and Simultaneous Integrated Boost (SIB) technique were used on 33 and 16 cases respectively, for treatment planning. Replanning took place in seven cases. All patients underwent FMISO PET/CT examinations before the start of radiotherapy (W0), and two consecutive FMISO examinations at the second (W2) and fifth week (W5) after the start of treatment. In detail, at W0 and W2, all patients (49) underwent FMISO examination, while 41 patients received a FMISO PET/CT scan at W5. The image acquisitions were planned at 160 min after the injection of 4 MBq per kg of body weight tracer, and for a duration of 10 min. The FMISO images were imported in our treatment planning system, Eclipse® version 15.6 (Varian Medical Systems, Inc., Palo Alto, USA), for subsequent analyses.

GTVs were delineated by board-certified radiation oncologists on the planning CT, based on FDG PET/CT and multi-parametric MRI (mpMRI) [34]. The expansion to clinical target volume (CTV) and planning target volume (PTV) was performed based on the standard operating procedure (SOP). The acquisition of both FDG and mpMRI took place before the start of chemoradiation.

The GTV, defined on the planning CT, represents the initial GTV before therapy. Subsequently, this initial GTV was transferred from the planning CT to W0-, W2- and W5-FMISO PET/CT imaging using deformable image registration of the corresponding CT volumes. This process was performed on Eclipse® Image Registration v15.5.

In our investigation, we focus on the hypoxic volume at W0, W2 and W5. The propagation of the initial GTV to W2 and W5, through deformable image registration, improved its allocation within the anatomical boundaries in W2 and W5 and accounted for anatomical changes due to differences in positioning, occurred tissue (tumour) shrinkage or weight loss.

The projected initial GTV on FMISO PET/CT in W2 and W5 does not accurately represent the true GTV at those time points. In our patient cohort, FDG PET/CT was available only at W0 and mpMRI was not available for all patients at W2 and W5. Consequently, delineating the “true” GTV at W2 and W5 was not feasible. These projected initial GTV volumes were utilised as a confining region for analysing SUV and HTV topography before and after the PVE correction.

The mean volume of the GTV across the entire patient cohort was 42.0 ± 42.9 ml (range 1.6–205.1 ml). The age of the group was 60 years on average, ranging from 34 to 78 years. Relevant details for our patient cohort are listed in Table 1.

**Table 1:** Characteristics of the patients included in our cohort

	Patients (N = 49)	
*Sex*		
Male	43	(88%)
Female	6	(12%)
*Stage*		
T1	1	(2%)
T2	8	(16%)
T3	15	(31%)
T4	25	(51%)
N0	4	(8%)
N1	2	(4%)
N2a	1	(2%)
N2b	13	(27%)
N2c	27	(55%)
N3	2	(4%)
*HPV*		
Positive	16	(33%)
Negative	33	(67%)
*Grading*		
G1	1	(2%)
G2	22	(45%)
G3	23	(47%)
Unknown	5	(10%)
*Region*		
Oral cavity	3	(6%)
Oropharynx	24	(49%)
Hypopharynx	9	(18%)
Larynx	5	(10%)
Multiple	8	(16%)

#### Application of the RC-based PVE correction on clinical data

**Fig. 3:**
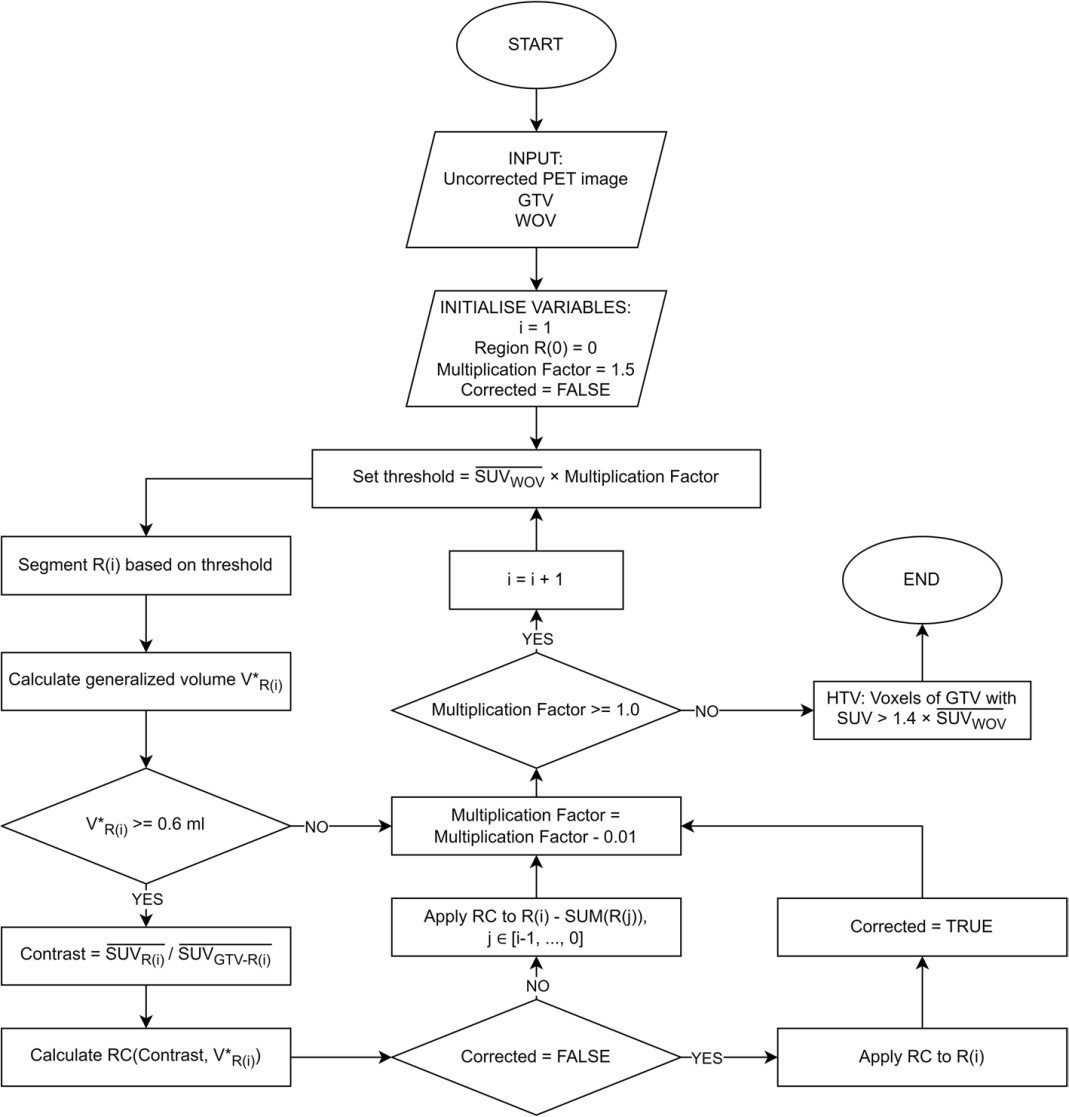
The flowchart describes the algorithmic process that was implemented to apply the RC-based PVE-correction method on the clinical data and to define the HTV based on corrected SUVs

The PVE correction was applied in a stepwise manner (see Fig. 3), targeting regions with specific SUV ranges by SUV-thresholding. At each step, a threshold generates a region of voxels or object. Since the specific RC for this object derives from Eq. 3, a volume for this object is required. To account for the irregular geometry of the object, a generalised volume V^*^ was defined. The generalised volume is given by [17]:

4 $$V^{*} = 36\pi \frac{{V_{object}^{3} }}{{S_{object}^{3} }}$$

where S_*object*_ and *V*_*object*_ are the surface area and volume of the object.

To set the starting threshold, a well-oxygenated volume (WOV) was initially defined. Typically, the aorta or a muscle tissue is used for this purpose. Since, in our PET/CT data, the aorta was outside the scanned region, muscle tissue was chosen as WOV [35]. Specifically, a portion of the sternocleidomastoid muscle, on the opposite side of the location of the GTV, was delineated as WOV by board-certified radiation oncologists, and the mean SUV ($\overline{{SUV_{WOV} }}$) was calculated.

A threshold of $\overline{{SUV_{WOV} }} \times 1.4$ is commonly used for defining HTV [19–21, 35]. A higher multiplication factor than 1.4 would generate more sub-regions within GTV. For our data and when applying thresholds above $\overline{{SUV_{WOV} }} \times 1.5$, V* was lower than the minimum volume (V ≥ 0.6 ml), used for fitting of the RC equations (Eq. 2). Therefore, the starting threshold (threshold-1) was set to $\overline{{SUV_{WOV} }} \times 1.5$. The voxels within the GTV, whose SUV superseded this threshold, defined region-1.

The RC(V*,C) for region-1 is given by Eq. 3, where V* is the generalised volume of region-1 and *C* is the contrast given by:

5 $$C = { }\frac{{\overline{{SUV_{{region{ }1}} }} }}{{\overline{{SUV_{GTV - region 1} }} }}$$

The SUVs of the voxels in region-1 were then corrected by dividing their original SUV by the calculated RC.

The next region, region-2, was generated by decreasing the multiplication factor by 0.01 to define threshold-2. Region-2 consisted of voxels with original SUV in the range between threshold-1 and threshold-2. The RC for region-2 is given by Eq. 3, with V* being the generalised volume of region-1 and region-2 combined and the corresponding *C*, defined according to:

6 $$C = { }\frac{{\overline{{SUV_{{region{ }1 + region{ }2}} }} }}{{\overline{{SUV_{{GTV - \left( {region 1 + region 2} \right)}} }} }}$$

The SUV for all voxels in region-2 was corrected by dividing their original SUV by the RC of region-2. This was repeated until the multiplication factor reached 1.0 and thus the threshold became $\overline{{SUV_{WOV} }} \times 1.0$. Finally, using the corrected SUVs inside GTV, the HTV was defined based on the threshold $\overline{{SUV_{WOV} }} \times 1.4$.

#### Evaluation of clinical data after PVE correction

A SUV analysis for GTV and HTV and a topographical comparison of HTV was performed for the PET/CT volumes before and after the PVE correction. The HTV was in each case generated based on the SUV threshold of $\overline{{SUV_{WOV} }} \times 1.4$. For the SUV analysis, the mean SUV in the GTV ($\overline{{SUV_{GTV} }}$) and HTV ($\overline{{SUV_{HTV} }}$) was used. In addition, the hypoxic fraction (HF) defined as the percentage of the GTV that is characterised as hypoxic:

7 $$HF = \frac{HTV}{{GTV}} \times 100 \%$$

was used to describe the extension of hypoxia and to compare the results before and after PVE-correction. The HF, by definition, can be calculated only in W0, since the actual GTV was not available at W2 and W5. For the topographical comparison of the generated HTVs, the volume, the DICE coefficient, the shift of centre of gravity (COG) and the Hausdorff distance were the used metrics.

### Test selection for statistical comparisons

The statistical comparison of two groups, before and after the PVE correction, was made with a paired, two-sided Wilcoxon signed-rank test since our data were not normally distributed. The significance level was set at 0.05.

## Results

### Calculation of recovery coefficients

**Fig. 4:**
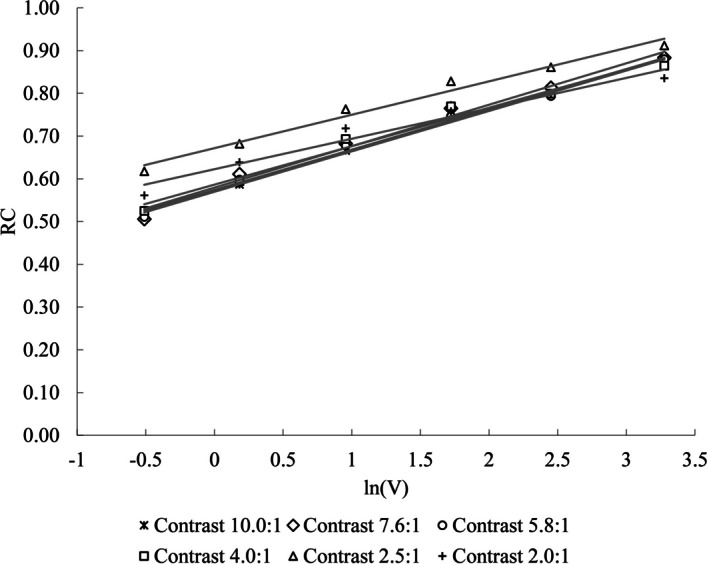
Graphical representation of the measured recovery coefficients and the fitted lines (Eq. 2) for different sphere volumes and contrasts as a function of volume *V*

The results of the measurements using phantom-1 for all six-contrast levels (Eq. 1) are shown in Fig. 4, where the results of fitting Eq. 2 to the experimental data are summarised in Table 2.

**Table 2:** Results of the semi-logarithmic linear regression fitting for RC in dependence on volume *V* (six spheres) for the six different contrast levels

Parameter	Contrast level					
	10.0: 1	7.6: 1	5.8: 1	4.0: 1	2.5: 1	2.0: 1
Intercept *b*	0.570 ± 0.006	0.579 ± 0.011	0.574 ± 0.008	0.586 ± 0.013	0.672 ± 0.010	0.623 ± 0.013
Slope *a*	0.095 ± 0.003	0.097 ± 0.006	0.094 ± 0.004	0.090 ± 0.007	0.078 ± 0.006	0.071 ± 0.007
*R*^*2*^	0.99	0.99	0.99	0.98	0.98	0.96

The representation used is value ± standard error. The coefficient of determination *R*^2^ is also listed. All parameter values are significant with p < 0.001

### Contrast-dependence of RC

**Fig. 5:**
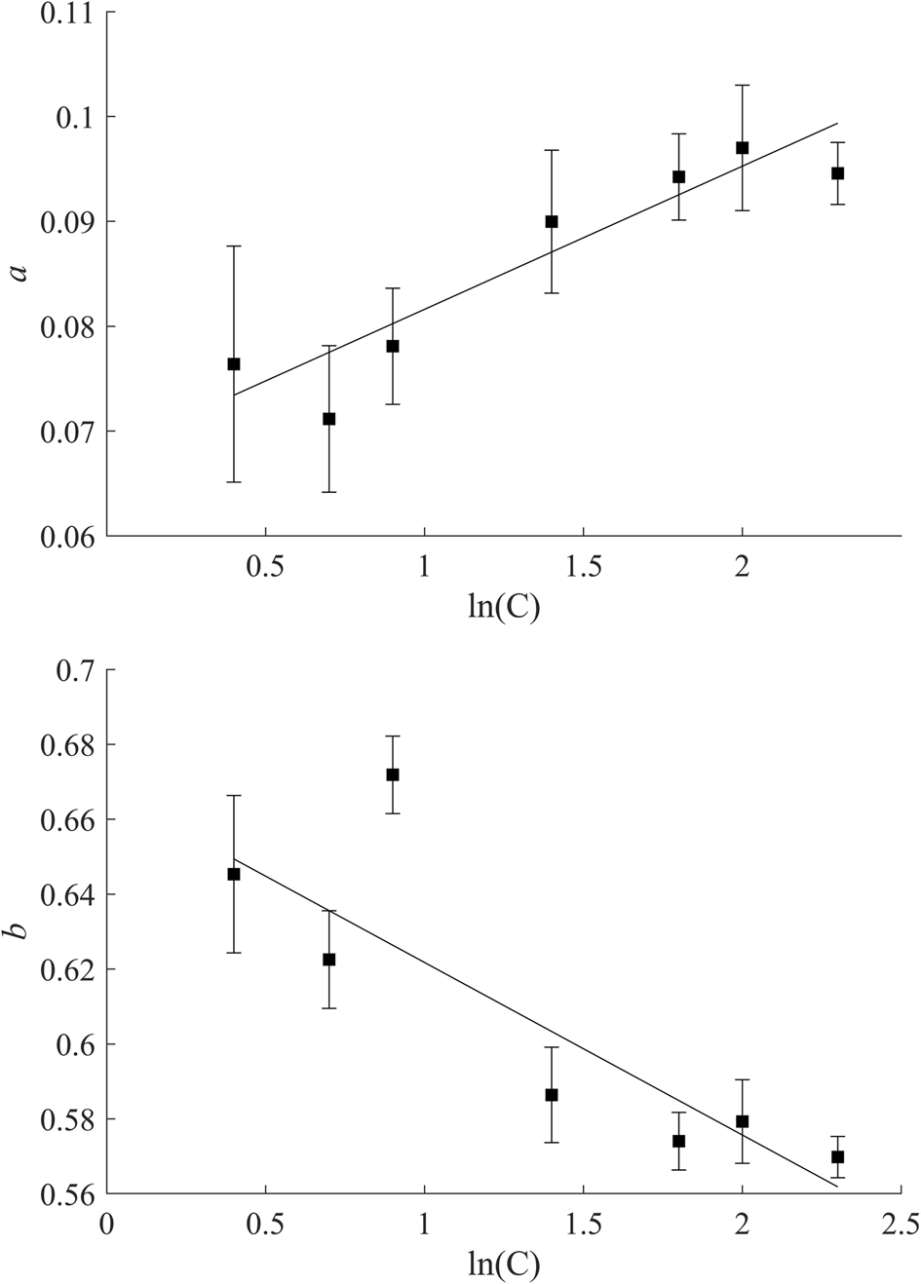
The semi-logarithmic weighted fitting graph of ***a*** and ***b***, relative to contrast. Data are shown with their standard error as listed in Table 2

The values of *a* and* b* listed in Table 2 are presented in the two semi-logarithmic graphs of Fig. 5.

Based on the representations in Fig. 5, a linear regression fit for the contrast-dependence of *a* and* b* was used:

8 $$a\left( C \right) = A_{a} \times \ln \left( C \right) + B_{a}$$

9 $$b\left( C \right) = A_{b} \times \ln \left( C \right) + B_{b}$$

with *A*_*a*_ = 0.014 ± 0.003 (*p* < 0.001), *B*_*a*_ = 0.068 ± 0.003 (*p* < 0.001) with *R*^*2*^ = 0.86 (*p* = 0.003) and.

*A*_*b*_ = − 0.046 ± 0.013 (*p* = 0.016), *B*_*b*_ = 0.668 ± 0.017 (*p* < 0.001) with *R*^*2*^ = 0.72 (*p* = 0.016). The fitting was done with the weighted least squares method, using the *fitlm()* function on MATLAB (9.9.0.1524771 (R2020b) Update 2, Natick, Massachusetts: The MathWorks Inc.). Finally, the general equation describing the volume- and contrast-dependence of RC (Eq. 3) becomes:

10 $$RC\left( {C,V} \right) = { }\left( {\left( {{\varvec{A}}_{{\varvec{a}}} \times ln\left( C \right)} \right) + {\varvec{B}}_{{\varvec{a}}} } \right) \times \ln \left( V \right) + \left( {\left( {{\varvec{A}}_{{\varvec{b}}} \times ln\left( C \right)} \right) + {\varvec{B}}_{{\varvec{b}}} } \right)$$

### Validation with inhomogeneous activity volumes

**Fig. 6:**
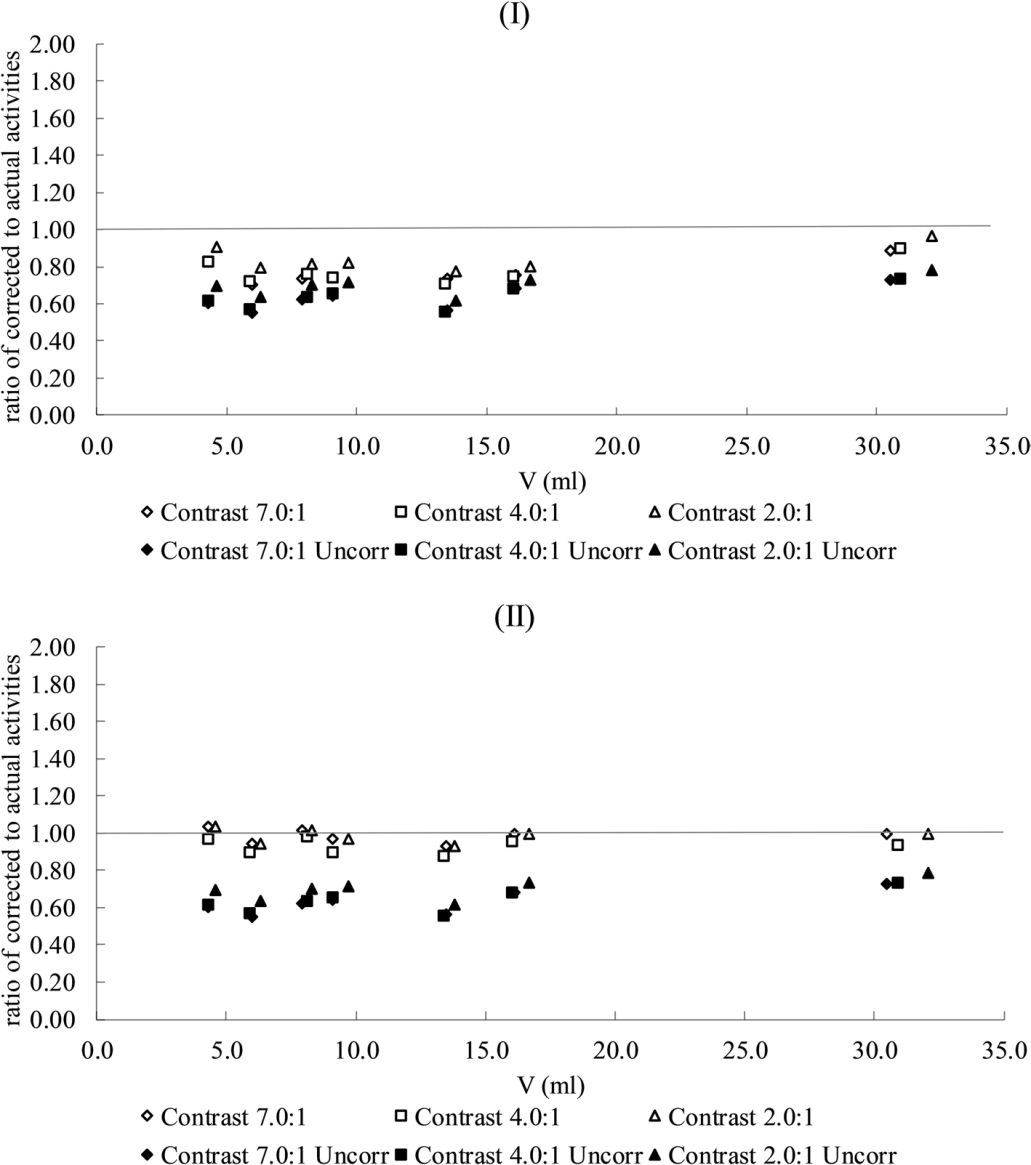
The ratio of corrected measured versus actual activity for the seven cylindrical ROIs and the three used contrasts. The RCs were calculated based on the cylinders’ volume (volume-method, **I**) or on the cylinders’ diameter (diameter-method, **II**). The ratio of uncorrected to actual activity is also shown for both methods

The RC-model (Eq. 10) was validated by using it to correct the acquired PET data using Phantom-2 (Fig. 2). For each contrast, the ratio of corrected to actual activities was analysed in relation to the lesion volume (Fig. 6).

As described previously, in the section about the validation with inhomogeneous activity volumes, the selected RC corresponds to the volume of sphere either with the same volume (Fig. 6I), or the same diameter as the cylindrical lesion under correction (Fig. 6II).

The mean ratio of the corrected measured activity to the actual activity for all seven cylindrical ROIs for the highest contrast 7:1 was 0.77 ± 0.06 (range of 0.70–0.88) and 0.95 ± 0.04 (range of 0.89–1.00) for the volume-based and the diameter-based correction method respectively. For the contrast 4:1, the ratio was 0.77 ± 0.06 (range of 0.71–0.90) for the volume-based method and 0.93 ± 0.04 (range of 0.88–0.98) for the dimeter-based method. For the lowest used contrast 2:1, the ratio was 0.84 ± 0.06 (range of 0.78–0.96) and 0.98 ± 0.03 (range of 0.93 to1.04) for the two correction methods respectively.

When no correction was applied to the measured activities (uncorrected), the mean ratio of measured to actual activity was 0.63 ± 0.06 (range of 0.55–0.73) for both 7:1 and 4:1 contrast levels and 0.70 ± 0.05 (range of 0.62–0.78) for the lowest 2:1 contrast.

The diameter-based correction method resulted to the best agreement between corrected and actual activity across all contrasts and volumes. The results of the validation proved that using simply the volume of a non-spherical object to calculate its RC is not adequate. Therefore, the generalised volume, based on the concept of generalised radius [17], which accounts for the influence of shape on PVE, has been implemented for the clinical data.

### Effect of PVE correction on clinical data

In this section, we describe how the detection of hypoxia and the uptake values in our clinical dataset are affected by the application of the PVE correction. The results are stated sequentially for W0, W2 and W5 and the only meaningful comparison is for each time point alone, before and after the application of the correction. Temporal comparisons are not appropriate since the anatomic and metabolic evolution of the tumour is affected by numerous factors and imaging corrections should not be correlated with such changes.

#### Effect of PVE-correction on individual PET/CT examination

In the available clinical data (49 cases for W0 and W2 and 41 for W5) and considering the original (uncorrected) FMISO SUVs, the cases with a present HTV (HTV > 0 ml) were 42 at W0, 39 at W2 and only 15 at W5. The $\overline{{SUV_{HTV} }}$ and the $\overline{{SUV_{GTV} }}$ after PVE-correction at all these three time points was significantly increased for every patient affected by the PVE-correction algorithm. Due to volume constraints, presented in the description of the PVE-correction method, the correction algorithm did not affect all cases. Specifically, all cases were affected at W0, 45 at W2 and 31 at W5.

After applying the PVE-correction to all cases, hypoxic volume (HTV > 0 ml) was detected in all cases at W0, in 45 at W2 and in 32 at W5. Thus, in all 7 patients (100%) with no hypoxia detectable before correction at W0, an HTV could be demonstrated after correction. Similarly, in 6 out of 10 cases (60%) at W2 and in 17 out of 26 patients (65%) at W5, hypoxia could be detected after correcting for PVE.

For the 7 patients with detectable hypoxia only after correction at W0, the average increase in $\overline{{SUV_{HTV} }}$ was 52% inside the HTV region, as segmented after the partial volume correction, and 27% in $\overline{{SUV_{GTV} }}$. For the 6 patients at W2, the corresponding increase was 54% and 29%, where for the 17 patients at W5, the increase amounted to 54% and 24%, respectively. The increase in $\overline{{SUV_{HTV} }}$ and $\overline{{SUV_{GTV} }}$ was statistically significant with *p* < 0.001 for each of these patients.

#### Effect of PVE-correction on SUV in HTV and GTV

**Fig. 7:**
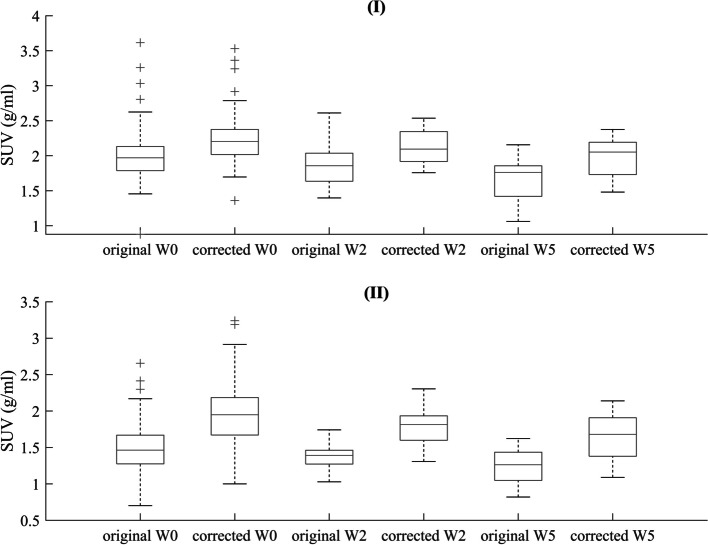
Graphical representation of the analysis of $\overline{{SUV_{HTV} }}$ (**I**) and $\overline{{SUV_{GTV} }}$ (**II**) for all patients and the three acquisitions, W0, W2, W5, before and after the PVE-correction

The analysis of $\overline{{SUV_{HTV} }}$ and $\overline{{SUV_{GTV} }}$ before and after applying the PVE-correction is shown in Fig. 7 using boxplots.

The population mean of $\overline{{SUV_{HTV} }}$, the standard deviation and the range (minimum to maximum value) for the uncorrected clinical data was 2.0 ± 0.5 g/ml (range of 0.9–3.6) g/ml at W0, 1.9 ± 0.3 g/ml (range of 1.4–2.6) g/ml at W2 and 1.7 ± 0.4 g/ml (range of 1.1–2.6) g/ml at W5. After correcting for PVE, the population mean of $\overline{{SUV_{HTV} }}$ was 2.2 ± 0.4 g/ml (range of 1.4–3.5) g/ml, 2.1 ± 0.2 g/ml (range of 1.5–2.5) g/ml and 2.0 ± 0.2 g/ml (range of 1.5–2.4) g/ml at W0, W2 and W5 respectively. The observed increase of $\overline{{SUV_{HTV} }}$ in the population mean, after PVE-correction by 10% at W0, 11% at W2 and by 18% at W5, was statistically significant with *p* < 0.001.

The population mean of $\overline{{SUV_{GTV} }}$ before correction was 1.5 ± 0.4 g/ml (range of 0.7–2.7) g/ml, 1.4 ± 0.2 g/ml (range of 1.0–1.7) g/ml and 1.2 ± 0.2 g/ml (range of 0.8–1.6) g/ml at W0, W2 and W5. After applying the PVE correction, it increased to 1.9 ± 0.4 g/ml (range of 1.0–3.2) g/ml, 1.7 ± 0.2 g/ml (range of 1.3–2.3) g/ml and 1.5 ± 0.3 g/ml (range of 1.0–2.1) g/ml respectively. Similarly to the HTV, the PVE correction resulted to a statistically significant increase in the population mean of $\overline{{SUV_{GTV} }}$ by 27% at W0, 21% at W2 and 25% at W5, with *p* < 0.001.

#### Effect of PVE-correction on HTV topography

**Fig. 8:**
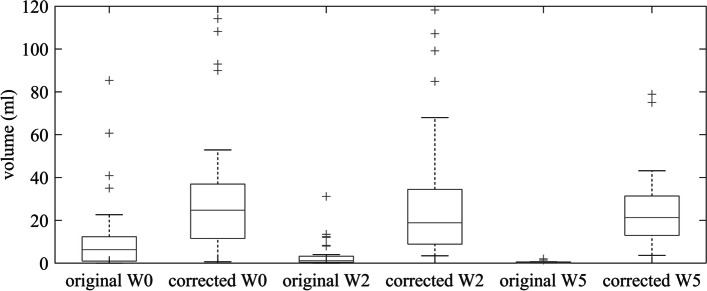
Graphical representation of the HTV volume for the three acquisitions at W0, W2 and W5, before and after the PVE correction

The volume of HTV before the PVE correction was on average 11.2 ± 16.8 ml (range of 0.0–85.4 ml) at W0, 3.5 ± 5.9 ml (range of 0.0–31.2 ml) at W2 and 0.4 ± 0.6 ml (range of 0.0–2.0 ml) at W5. After the PVE correction, the mean value of HTV volume was increased to 30.3 ± 33.0 ml (range of 0.6–155.1 ml), 27.2 ± 28.8 ml (range of 2.7–118.2 ml) and 18.7 ± 19.7 ml (range of 1.3–78.9 ml) respectively. Comparing the HTV defined before and after applying the PVE correction, there was an enormous increase in the volume of HTV of the population by an average of 171% at W0, 691% at W2 and approximately 4.60 × 10^3^% at W5, all statistically significant with *p* < 0.001. Figure 8 demonstrates graphically the comparison of the HTV volume before and after the correction.

The mean value of the DICE similarity coefficient for the HTV before and after the correction was 0.4 ± 0.3 (range of 0.0–1.0) at W0, 0.2 ± 0.2 (range of 0.0–0.7) at W2 and 0.03 ± 0.04 (range of 0.00–0.14) at W5. This is in-line with the observed significant increase in HTV volume after applying the PVE-correction. The mean Hausdorff distance was 23 ± 10 mm (range of 0–43 mm), 28 ± 10 mm (range of 8–56 mm) and 39 ± 14 mm (range of 22–72 mm) at the three time points respectively. Considering the mean distance between the centres of gravity, this was 6 ± 4 mm (range of 0–19 mm) at W0, 8 ± 6 mm (range of 1–33 mm) at W2 and 13 ± 8 mm (range of 2–34 mm) at W5. We observed a consistent significant reduction of DICE and significant increase of Hausdorff distance and shift of COG with progression of time during the radiotherapy.

The HF (Eq. 7) was originally 18.4 ± 18.4% (range of 0.0–60.3%) at W0. After the PVE-correction, HF increased to 69.9 ± 20.0% (range of 8.8–99.9%) at W0. Thus, HF increased on average by 280% with *p* < 0.001.

## Discussion

The calculation of the RCs showed that the PVE caused a severe underestimation of SUV, especially in low-volume and low-contrast lesions where RC was as low as 18% at contrast level 2:1. Thus, the need for PVE correction was necessary. The proposed PVE correction method is tailored for use across low contrast tracers, such as FMISO and was firstly successfully validated on inhomogeneous activity distribution set-up with non-spherical lesions and then to our clinical data.

Furthermore and for each individual patient, we could demonstrate a statistically significant increase in $\overline{{SUV_{HTV} }}$ and $\overline{{SUV_{GTV} }}$ after applying the PVE-correction. For patients with no detectable hypoxia, after applying the PVE-correction, a hypoxic volume could be identified in 100% of them at W0, in 60% at W2 and in 65% at W5.

In the population-based analysis, it was demonstrated that the PVE correction resulted in a 10–18% increase in $\overline{{SUV_{HTV} }}$ and a 21–27% increase in $\overline{{SUV_{GTV} }}$. The PVE correction also induced an extreme topographical change in HTV, something clearly indicated by the increase in the volume of HTV up to a factor of 47, the high Hausdorff distance values (range of 23–39 mm for the population mean) and the large COG-shifts (range of 6–13 mm for the population mean).

In this study, certain choices were made, that influenced the presented results. First of all, the selection of the RC-based method for the correction of the PVE. It is a relative simple method and can be easily implemented in a clinical environment. However, this method applies local corrections, which is only acceptable in cases where a globally corrected image is of no importance, as it is in our case, where the region of interest is confined inside the GTV.

Furthermore, the determination of RC in the phase of the PET/CT scanner modelling depends highly on the accuracy of the segmentation. This is a limitation that does not affect global correction methods, which themselves come with their own assumptions and limitations. Firstly, reconstruction-based correction requires the raw data (sinograms) to which we did not have access. Image-based correction methods and in particular, iterative deconvolution methods (post-reconstruction), such as the ones proposed by Lucy-Richardson [36] and Van Cittert [37], do not take regional variations into consideration and assume uniform distribution of the activity, even when applied locally. They also amplify image noise and artefacts during the iterative deconvolution procedure.

A region-based correction method that was introduced by Hofheinz et al. [38] was also considered. This method however is developed for FDG PET data and focuses on the correction of the mean activity in the tumour. It assumes well-defined borders between two distinct regions, the tumour with high uptake and its surrounding normal tissue (background) with low uptake, and a high, expected contrast between them. This is not the case with tissue oxygenation since there are no histological boundaries between hypoxic and normoxic tissues, defined by a high contrast in uptake between them. In addition, the method proposed by Hofheinz is strongly reliant on one predefined boundary. The boundary does not change nor are new regions defined. Thus, it does not improve hypoxia detectability, which is the major concern in our study. On the other hand, the RC-based correction was adjusted to operate iteratively, with the capacity to define new regions at each iteration.

Based on the measurements with spheres of homogenous activity, RCs were calculated from the mean and not the maximum activity value in the spheres. When applying this correction to a region of inhomogeneous activity distribution, on clinical data, the voxels in the centre of the region with higher SUV, might be overcorrected. On the other hand, if the maximum activity value was used to calculate the RCs, it is expected to have an undercorrection of the voxels with lower SUV, those voxels close to the borders of the region. Selecting either the mean or the maximum SUV to calculate the RCs, depends on the use case. In our study, it is more important not to undercorrect and thus not to underestimate hypoxia anywhere, so we decided for the mean SUV-based calculation of RC.

The calculation of RC requires the calculation of the mean activity in the glass spheres and this in turn requires an accurate segmentation of the region inside the glass on the CT images. When transferring the contour to the registered PET acquisition, especially for small spherical volumes, the contours were not perfectly shaped or positioned. This happened because of the differences in the resolution of the two modalities and could have lead to a miscalculation of the mean activity in the sphere and consequently of the RC. Thus, a supervision by the operator was required.

The original FMISO PET data demonstrated low HF values with a mean of 18.4 ± 18.4% and range of 0.0–60.3% at W0, significantly lower than those referred in the literature. Thorwarth et al. [39] reported for a cohort of 12 patients using FMISO PET, HF values with an average of 45.3 ± 35.5% and range of 0.3–95.7% for an average FDG-based GTV of 29.6 ± 19.2 ml. Thorwarth et al. used the same multiplication factor of 1.4 to set the threshold for the definition of HTV, as in our study.

Mönnich et al. [26], for a group of 21 patients, also using a multiplication factor of 1.4, published HF values with an average of 42.5 ± 36.6% and a range of 0.0–97.3% for an average FDG-based GTV of 12.8 ± 6.7 ml. For a smaller cohort of 6 patients and using also a multiplication factor of 1.4, Simoncic et al. [40] published lower HF values; mean value of 30.5 ± 24.9% and range of 1.0–72.0% for an average FDG-based GTV of 28.5 ± 12.2 ml.

Finally Nehmeh et al. [41] published for a cohort of 20 patients, even higher HF values with an average of 60.0 ± 36.1% and a range of 0.3–100.0% for an average FDG-based GTV of 9.8 ± 7.2 ml. The authors used a lower value for the multiplication factor (1.2), which could explain the higher calculated HF values in their investigation.

The calculated hypoxia after applying the PVE-correction to our 49 cases resulted to an average HF of 69.9 ± 20.0% and a range of 8.8–99.9% at W0. Although, the resulting average HF aligns better with the previously discussed published data, directly comparing HF values among different research institutions can be challenging. The cited publications present FDG-based segmentations of GTV, whereas our GTV segmentation is based on both FDG and mpMRI (GTV_total_ = GTV_FDG_ ∪ GTV_mpMRI_), resulting in a potentially higher tumour volume. Furthermore, the choice of the reference region, whether it is muscle tissue or arterial blood, influences the hypoxia-indicating threshold and, consequently, the volume of HTV. The applied corrections during reconstruction, PVE intensity and the strategy for its compensation may vary across different PET/CT scanners and research institutions.

Another limitation of our method, which should also be taken into consideration, is the uniformity of the activity distribution in the background. It was apparent in the experimental phantom measurements for the RC calculation and the validation of the PVE correction method, but not in the clinical data. The lack of background uniformity influences the PVE since the spill-out and spill-in effects are not isotropic.

In addition, tracers such as FMISO are used with the assumption that the absence of the tracer means normoxic conditions and presence of oxygen, but this is not always the case. In areas with very little to no blood flow, such as necrotic tumour regions, there is low to no presence of the tracer. The presence of such regions within the GTV can result to incorrect estimation of background for the PVE-correction method.

To conclude, we have presented an effective retrospective PVE-correction, based on RC and adapted for low-contrast PET tracers, such as FMISO, which can be easily reproduced at other PET/CT scanners, following the described methodology. It is advised to apply the correction on clinical data with caution, always taking into consideration the aforementioned limitations and assumptions.

## Data Availability

The datasets during and/or analysed during the current study are available from the corresponding author on reasonable request.

## Abbreviations

BG: Background
CRT: Chemoradiotherapy
CT: Computation tomography
FMISO: [^18^F]fluoromisonidazole
GTV: Gross tumour volume
HF: Hypoxic fraction
HNSCC: Head and neck squamous cell carcinoma
PET: Positron emission tomography
PMMA: Polymethylmethacrylate
PVE: Partial volume effect
RC: Recovery coefficient
ROI: Region of interest
SIB: Simultaneous integrated boost
SOP: Standard operating procedure
SUV: Standardised uptake value
WOV: Well-oxygenated volume
